# Melanocortin-3-receptor promoter polymorphism associated with tuberculosis susceptibility does not influence protein expression

**DOI:** 10.1186/1756-0500-6-99

**Published:** 2013-03-15

**Authors:** Marlene Eggert, Martina Pfob, Ortrud K Steinlein

**Affiliations:** 1 Institute of Human Genetics, University Hospital, Ludwig-Maximilians-University Munich, Munich, Germany

**Keywords:** Melanocortin-3-receptor, MC3R, Polymorphism, Tuberculosis susceptibility, Luciferase reporter assay

## Abstract

**Background:**

The melanocortin-3-receptor (*MC3R*) is a member of the G-protein coupled receptor family that mediate cellular response through the cyclic adenosine monophosphate signalling pathway. In the promoter region of *MC3R* the polymorphism rs6127698 has previously been shown to be strongly associated with tuberculosis susceptibility. It is predicted to generate an alternative transcription factor binding site.

**Findings:**

We investigated the functional impact of rs6127698 by luciferase assay to assess if this polymorphism is capable of altering protein expression. Our results did not show any significant protein expression changes when comparing the two alleles of rs6127698.

**Conclusions:**

Our experiments demonstrate that the rs6127698 polymorphism does not influence protein translation. A functional role of the predicted alternative transcription factor binding site could therefore not be confirmed. These results suggest rs6127698 has no direct role in tuberculosis susceptibility. The possibility remains that this polymorphism is linked to an adjacent functional genetic variant, acting as a surrogate marker for disease risk.

## Findings

### Introduction

Tuberculosis still poses a severe health problem worldwide and is classified as the second most frequently cause of death in respect of infectious diseases according to the World Health Organization [1]. Great effort has been made to find genomic regions and specific genes within these regions that play a role in tuberculosis susceptibility, respectively in protection against the disease [2-4]. Cook et al. found two genomic loci linked to tuberculosis susceptibility by sibling pair analysis and could further narrow it down to two genes, one of them being the melanocortin-3-receptor gene (*MC3R*) [2]. This G-protein coupled receptor has been implicated in a broad spectrum of physiological processes such as energy homeostasis, fat metabolism and immune response [5,6]. In a recently performed study by Adams et al. the major allele guanine (G) of the single nucleotide polymorphism (SNP) rs6127698 in the promoter of *MC3R* showed a highly significant association with tuberculosis susceptibility. In this work, an *in silico* predicted alternative transcription binding site generated by the G allele was discussed as a possible reason for the association with tuberculosis [7]. The aim of the present study was to investigate the hypothesis that the G allele of SNP rs6127698 is able to regulate protein expression due to the creation of a transcription factor binding site.

### Material and methods

Firefly luciferase vector pGL4.10 (AY738222.1) and renilla luciferase vector pGL4.74 (AY738230.1) were purchased from Promega (Mannheim, Germany). The promoterless vector pGL4.10, encoding the firefly luciferase reporter gene luc2, contains a multiple cloning site upstream of luc2 to enable promoter studies. Vector pGL4.74, harbouring an HSV-TK promoter and the renilla luciferase reporter gene hRluc, is used as an expression control to normalize for transfection differences. The promoter region and 5^′^ untranslated region (5^′^UTR) from position −1 to −984 of the *MC3R* gene (NM_019888.3) were synthesized (MWG Eurofins, Ebersberg, Germany) with the endogenous KpnI restriction site mutated and cloned into the multiple cloning site of pGL4.10 with KpnI and NcoI (Fermentas, St. Leon-Rot, Germany). After cloning, the correct sequence with the endogenous KpnI restriction site was restored by site-directed mutagenesis and confirmed by sequencing. The minor SNP allele T was also created by site-directed mutagenesis.

The human embryonic kidney cells (HEK) 293 were cultured in T75 flasks in monolayer with DMEM (Sigma-Aldrich, Hamburg, Germany) containing 4.5 g glucose, 10% FBS, 1% L-Glutamine and 1% Penicillin/Streptomycin (Invitrogen, Karlsruhe, Germany) and maintained in a humidified atmosphere at 37°C and 5% CO_2_. For the experiments, the cells were co-transfected with the reporter plasmid pGL4.10 containing the *MC3R* insert with the SNP allele G (*MC3R*-G) or the SNP allele T (*MC3R*-T), respectively, and the control plasmid pGL4.74 at a ratio 20:1 using TransIT ®-LT1 Transfection Reagent (MoBiTec, Göttingen, Germany) with 24 h transfection prior to luciferase assay. The cells were seeded 24 h prior to transfection with 3x10^5^ cells in 24 well plates (Greiner bio-one, Frickenhausen, Germany). The firefly and renilla luciferase activities were measured with the TRiStar LB941 (Berthold Technologies, Bad Wildbad, Germany) by the Dual-Glow® luciferase assay system (Promega, Mannheim, Germany) according to the manufacturer’s protocol. The ratio of firefly luciferase and renilla luciferase activity was determined and normalized to pGL4.10. The various 5^′^UTR constructs are expressed as fold change to pGL4.10.

The experiment was repeated independently three times with triplicate samples. A two-tailed *t*-test was used to compare the values of the test samples and the control samples. A *p* value of *p* < 0.05 was considered statistically significant. The data are expressed as fold change and as mean ± SEM.

### Results and conclusion

**Figure 1:**
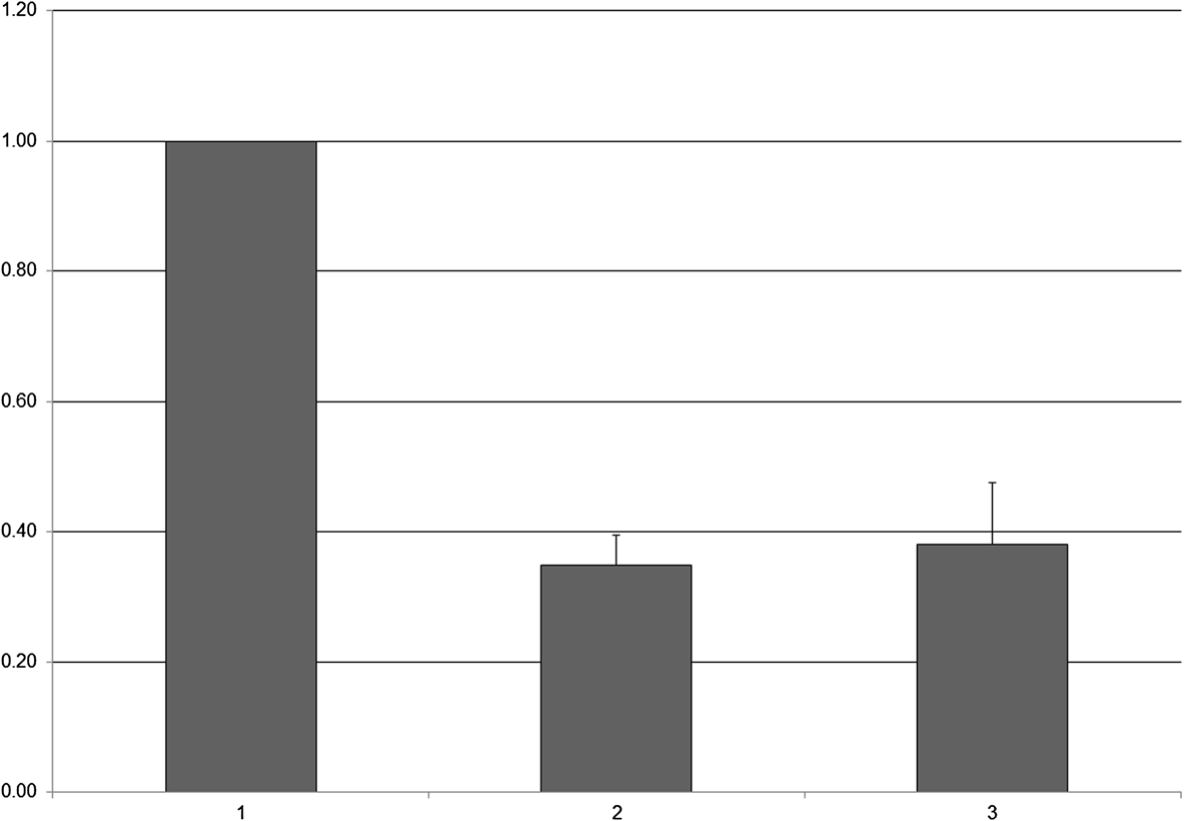
**No altered protein expression was observed when comparing the two alleles of SNP rs6127698 in the promoter region of *MC3R. *** Fold change of firefly activity normalized to pGL4.10, is illustrated for the three different constructs; 1, pGL4.10; 2, *MC3R*-G; 3, *MC3R*-T.

The two constructs *MC3R*-G and *MC3R*-T were compared to each other by luciferase assay. The results showed no significant differences in protein expression between the two SNP allele variants (*MC3R*-G 0.35 ± 0.05; *MC3R*-T 0.38 ± 0.09; *p* = 0.816) (Figure 1). Thus, our findings do not support the previously reported hypothesis that the major allele G of SNP rs6127698 creates an alternative transcription binding site [7]. At least in our experimental setting, rs6127698 did not show any significant impact with regard to *MC3R* promoter function. However, we cannot rule out the possibility that that the predicted transcription factor binding site created by the major allele G only becomes functional in certain cell types. Such a phenomenon has been reported in the literature before, e.g. for a transcription site in the human glutathione transferase kappa promoter [8]. Cell-specific transcription factors or other non-ubiquitously expressed proteins might be possible reasons for such a selective mechanism. Furthermore, we cannot exclude the possibility that the major allele G of SNP rs6127698 initiates a different, yet unknown, biological mechanism not detectable by the methods used here. Such an unknown mechanism could account for the reported association. A third possibility would be that the SNP allele itself might be non-functional but linked to a nearby polymorphic site that itself affects protein expression or function. Further research is needed to elucidate the precise role of *MC3R* and its variants in terms of tuberculosis susceptibility.

## Competing interests

The authors declare that they have no competing interests.

## Authors’ contributions

ME designed the study, carried out the experiments, analyzed the data and drafted the manuscript. MP helped to perform the experiments, to acquire the data and to draft the manuscript. OS designed the study, interpreted the data and critically revised the manuscript. All authors have given their final approval of the submitted version.
